# Spontaneous breathing trial reduces mechanical ventilation weaning when compared with SmartCare™ ventilation

**DOI:** 10.1186/cc12659

**Published:** 2013-06-19

**Authors:** C Taniguchi, KT Timenetsky, CSM Silva, E Giovanetti, R Henn, RAC Eid, CSV Barbas

**Affiliations:** 1 Albert Einstein Jewish Hospital, Morumbi, São Paulo, SP, Brazil

## Introduction

Mechanical ventilation (MV) weaning is commonly performed using a spontaneous breathing trial (SBT) with pressure support ventilation after a daily weaning screen [1]. Recently there has been increased interest in automatic weaning trials, using respiratory rate, tidal volume and ETCO_2 _monitoring during SBT [1,2]. So far there is no clinical evidence comparing an automatic weaning trial with SBT. Our study's objective was to compare MV and weaning times between Automatic Weaning Ventilation System (SmartCare™/PS) and SBT groups.

## Methods

A randomized, controlled study was performed in a general ICU. We enrolled adult patients who were ventilated for more than 24 hours. Patients were randomized either to the control or SmartCare™ group. All patients were ventilated with a Drager EvitaXL (Drager Medical, Lubeck, Germany) ventilator with SmartCare™/PS software version 1.1. The control group consisted of a daily weaning screen and SBT with pressure support ventilation; if patients tolerated SBT they were extubated. SmartCare™ group patients were also submitted to a daily weaning screen, after which they were ventilated with the SmartCare™/PS mode. We evaluated the MV and weaning time, maximum inspiratory pressure, maximum expiratory pressure, vital capacity, respiratory frequency to tidal volume ratio (f/Vt), use of noninvasive ventilation (NIV) post extubation, and re-intubation rate.

## Results

We evaluated a total of 70 patients (35 patients randomized in each group). There was no difference in age (*P *= 0.298) or gender (*P *= 0.08) between groups (Table 1). There was no difference in MV time between the control and SmartCare group (*P *= 0.534) (Table 1). Weaning duration was lower in the control group (pf/VT, *P *= 0.414), use of NIV post extubation (*P *= 0.811) and re-intubation rate (*P *= 1.0) (Table 1).

**Table 1:** Characteristics of patients between SmartCare™ and control groups

	SmartCare™	Control	*P *value*
Idade	60 (46 to 77)	65 (57 to 81)	0.298
Gender, male	23 (65)	16 (45)	0.07
MIP	45 (40 to 53)	40 (36 to 50)	0.272
MEP	40 (30 to 59)	40 (21 to 44)	0.059
VC	1,200 (900 to 1,850)	1,000 (500 to 1,600)	0.834
f/Vt	35 (24 to 55)	40 (26 to 68)	0.414
MV time	4 (2 to 6)	3 (2 to 7)	0.534
Weaning time	110 (80 to 120)	60 (50 to 80)	<0.001
Use of NIV post extubation	18 (51.4)	16 (45.7)	0.811
Reintubation rate	2 (5.7)	2 (5.7)	1.00

Data presented as median (interquartile range) or *n *(%). f/VT, respiratory rate to tidal volume ratio; MEP, maximum expiratory pressure; MIP, maximum inspiratory pressure; MV, mechanical ventilation; NIV, noninvasive ventilation; VC, vital capacity. *P value significant <0.05.

## Conclusion

SBT showed a reduction in weaning time when compared with the SmartCare™/PS group, although there was no impact on total MV time and reintubation rate.
